# Biomechanics of World-Class 800 m Women at the 2017 IAAF World Championships

**DOI:** 10.3389/fspor.2022.834813

**Published:** 2022-04-14

**Authors:** Brian Hanley, Stéphane Merlino, Athanassios Bissas

**Affiliations:** ^1^Carnegie School of Sport, Leeds Beckett University, Leeds, United Kingdom; ^2^International Relations and Development Department, World Athletics, Monte Carlo, Monaco; ^3^Athletics Biomechanics, Leeds, United Kingdom; ^4^School of Sport and Exercise, University of Gloucestershire, Gloucester, United Kingdom

**Keywords:** coaching, elite-standard athletes, kinematics, speed, track and field

## Abstract

The 800 m race challenges the aerobic and anaerobic energy systems, and athletes adopt a technique that allows for running efficiency as well as sprinting speeds. The aim of this novel study was to compare important kinematic variables between the two laps of the 2017 IAAF World Championships women's final. Video data (150 Hz) were collected of all eight finalists on both laps at a distance approximately 50 m from the finish line along the home straight. Running speed, step length, cadence, temporal variables, sagittal plane joint angles, and shank angle at initial contact were measured. Running speed was faster on lap 2 (*p* = 0.008) because of large increases in cadence (*p* = 0.012). These higher cadences resulted in large decreases in contact times (*p* = 0.031) and in flight times (*p* = 0.016) on lap 2. Greater knee flexion and ankle plantarflexion (*p* ≤ 0.039) at initial contact on lap 2 seemed partly responsible for shorter swing times (*p* = 0.016), and which accompanied a decrease in shank angle at initial contact from lap 1 (7°) to a more vertical position on lap 2 (4°) (*p* = 0.008). Coaches should note that the need for higher cadence, horizontal impulse production during shorter contact times, and the adoption of forefoot striking require strength and neural system training to allow for athletes to increase cadence during the sprint finish. Increasing cadence (and not step length) was the driving factor for faster finishing speeds in the women's 800 m.

## Introduction

The 800 m race is the shortest middle-distance track race held in major events such as the Olympic Games and World Athletics Championships, with outdoor competitions held over two 400-m laps. Racing over 800 m elicits both aerobic metabolism to its maximum power and anaerobic metabolism to its maximum capacity (Billat et al., 2009). Elite 800 m runners tend to adopt seahorse-shaped pacing (Casado et al., 2021), whereby a very fast start is followed by the slowest part of the race (between 200 and 400 m) and then a gradual increase in speed until the last 100 m. One interesting aspect of 800 m racing is thus that the athletes try to run their fastest speeds during the endspurt, when they are likely to be tiring and fatigue resistance is crucial (Haugen et al., 2021). The manner in which the 800 m is raced means that, in physiological terms, the two laps are quite different (Gamboa et al., 1996), and an appreciation of what mechanical changes occur between the first and second laps would therefore be valuable for athletes and coaches.

The two main factors in running speed are step length and cadence (also known as step rate or frequency); it follows that an athlete can only run faster if one or both of these spatiotemporal elements is increased. Cadence has been suggested to be a greater differentiator of running speed when accelerating to maximal speeds, not least because of how step length is limited by leg length (Hunter et al., 2004), itself affected by overall body height. Because higher cadences require the recruitment of a greater proportion of less efficient fast-twitch type II muscle fibers (Brisswalter et al., 2000), it follows that increasing cadence is a mechanical tactic best left to near the end of the race, when running economy can be sacrificed for speed (Bushnell and Hunter, 2007). Changes in cadence can lead to reduced contact time, although this could affect the magnitude of forward impulse required to achieve fast running speeds (Weyand et al., 2010), or to a reduction of flight time, which in turn could affect the distance achieved during flight and thereby reduce step length. A shorter contact time is associated with faster running because it results in a smaller duty factor (the proportion of a leg's cycle time spent in contact) (Forrester and Townend, 2015), which manifests as a “bouncy” running style where high vertical stiffness aids performance (Burns et al., 2021; van Oeveren et al., 2021). Maintaining short contact times is a key determinant of maintaining performance during runs to exhaustion (Hayes and Caplan, 2014), and highlights the value of analyzing temporal variables as athletes sprint to the finish in 800 m racing. However, no previous research has been conducted on world-class female 800 m runners in competition to see what changes are adopted in practice.

Changes in cadence result in changes in joint kinematics, with the knee most involved through an increase in flexion at initial contact, with an accompanying increase in ankle plantarflexion (Heiderscheit et al., 2011) and a decrease in shank angle at initial contact (Preece et al., 2019). These joint positions are associated with forefoot or midfoot striking, which is the predominant footstrike pattern amongst middle-distance runners (Hayes and Caplan, 2012), and is similarly found amongst the world's best male 10,000 m runners (Hanley et al., 2021b), possibly because of a potential association with better running economy (Santos-Concejero et al., 2014). Better long-distance runners were found to have smaller shank angles at initial contact (Folland et al., 2017; Preece et al., 2019); however, the participants in these studies were not elite athletes running at speeds found in championship racing. Given that middle-distance runners are predominantly forefoot or midfoot strikers (Hayes and Caplan, 2012), it could be assumed that their shank angles at initial contact would be close to vertical (ankle directly below or even behind the knee), but whether changes occur in joint or shank angles with increases (or decreases) in speed amongst elite middle-distance running has not been established. Such information could be useful to coaches with regard to developing a technical model of running for their athletes.

Much of what we know about running mechanics stems from research on longer distances, non-elite athletes, male runners only, or treadmill and other laboratory-based studies (Cunningham et al., 2013; Hanley et al., 2021b; Trowell et al., 2021), which might not reflect what occurs in the highly ecologically valid setting of a world-class competition. Indeed, many of these studies used set speeds that are not indicative of what occurs when athletes are racing to achieve the best possible finishing position. From a biomechanical viewpoint, an understanding of how world-class athletes alter their gait between slower and faster race paces is required to satisfactorily identify key biomechanical variables for athletes and coaches to develop in training. The aim of this observational study was to analyze changes in spatiotemporal and joint kinematic factors in world-class women's 800 m running between the end of the first and second laps. Based on previous research, it was hypothesized that athletes would run faster in the second lap because of greater step lengths and cadences, with corresponding changes to related spatiotemporal and joint kinematic variables.

## Materials and Methods

### Research Approval

Data were collected as part of the London 2017 IAAF World Championships Biomechanics Project. The use of those data for this study was approved by the IAAF (since renamed World Athletics), who own and control the data, and locally the study was reviewed and approved by Carnegie School of Sport Research Ethics Committee. The participants provided their written informed consent to participate in this study. The study was conducted in accordance with the recognized ethical standards of the Declaration of Helsinki.

### Participants

Eight athletes (age: 26 ± 3 years; stature: 1.73 ± 0.06 m; mass: 61 ± 6 kg) were analyzed approximately halfway along the home straight on the first and second laps of the women's 800 m final (~350 and 750 m of total race distance). The temperature at the start of the race was 22°C and the relative humidity was 35% (Hanley et al., 2018). Athletes' personal best (PB) and finishing times were obtained from the open-access World Athletics website (World Athletics, 2021a). Participants' dates of birth were obtained from World Athletics (2021b), whereas their body heights and masses were obtained from Matthews (2017) and online sources (en.wikipedia.com/wiki).

### Data Collection

**Figure 1:**
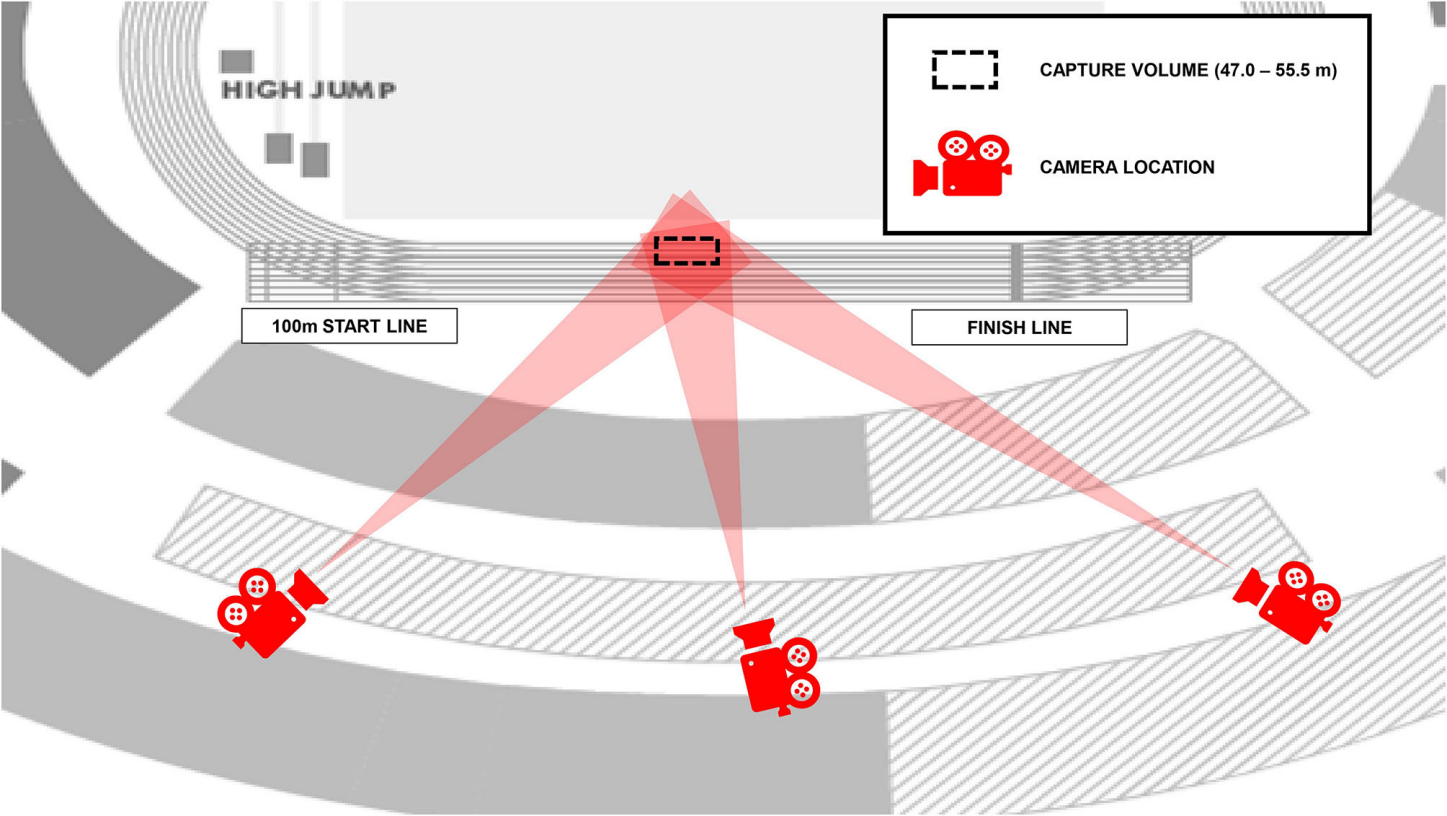
Camera positions for recording the women's 800 m final (laps 1 and 2) at the 2017 IAAF World Championships. The position on the track where the athletes were recorded is shown by the dashed lines.

Three stationary Sony PXW-FS7 digital cameras (Sony, Tokyo, Japan) recording at 150 Hz (shutter speed: 1/1,250 s; ISO: 1600; FHD: 1920 × 1080 px) were used to record the athletes as they ran through the calibrated middle section of the home straight (47.0–55.5 m from the start line used for the 100 m event). The cameras were placed in three locations on a broadcasting balcony along the home straight, and angled at approximately 45°, 100°, and 135° to the plane of motion, respectively (Figure 1). A rigid cuboid calibration frame (length: 3.044 m, width: 3.044 m, height: 3.044 m) was positioned twice over discrete predefined areas in the first two lanes on the running track to ensure an accurate definition of a volume within which all athletes ran. Markings on the frame produced 24 non-coplanar control points per individual calibrated volume (48 points in total) and facilitated the construction of a global coordinate system.

### Data Analysis

The collected video files were imported into SIMI Motion (version 9.2.2, Simi Reality Motion Systems GmbH, Germany) for analysis. An event synchronization technique (synchronization of four critical instants: right foot initial contact, right foot toe-off, left foot initial contact and left foot toe-off) was applied to synchronize the two-dimensional coordinates from each camera. The magnification tool in SIMI Motion was set at 400% to aid identification of body landmarks. The Direct Linear Transformation (DLT) algorithm (Abdel-Aziz et al., 2015) was used to reconstruct the three-dimensional (3D) coordinates from each camera's x- and y-image coordinates. The obscuring of many body segments in most athletes, caused by the tight “bunching” of the group, precluded a full-body digitizing process. Running speed was thus calculated by digitizing the athlete's head as a proxy for the center of mass (CM) (Hanley et al., 2021a), whereas distances and angles were calculated using 3D coordinate data found using the 3D still image measurement tool in SIMI Motion.

Descriptions of the spatiotemporal and joint angular variables analyzed in this study are presented in Table 1. Joint angular data were averaged between left and right sides, rounded to the nearest integer, and have been presented in this study at initial contact (defined as the first visible instant during stance where the athlete's foot clearly contacted the ground). Footstrike patterns were defined using the foot position at initial contact as either rearfoot (the heel contacted the ground first without simultaneous contact by the midfoot or forefoot), midfoot (the heel and midfoot contacted the ground together) or forefoot (the forefoot/front half of the sole contacted the ground first with a clear absence of heel contact) (Hasegawa et al., 2007). Two successive steps per athlete per lap were analyzed.

**Table 1:** Variables analyzed in the study and their description.

**Variable name**	**Description**
Running speed (km/h)	The mean CM horizontal speed during a complete gait cycle
Step length (m)	The distance between successive foot contacts from a specific event on the gait cycle on a particular foot (e.g., toe-off) to the equivalent event on the other foot
Cadence (Hz)	Calculated by dividing horizontal speed by step length (Mero and Komi, 1994)
Contact time (s)	The time duration from initial contact to toe-off
Flight time (s)	The time duration from toe-off of one foot to initial contact of the opposite foot
Swing time (s)	The time duration from toe-off on one foot to initial contact on the same foot
Duty factor	The proportion of stride time (contact time plus swing time) when the foot is in contact with the ground
Shank angle (°)	The angle between the lower leg and the ground, where 0° indicates a vertical position (knee directly above the ankle); angles greater than 0° indicate the ankle landed in front of the knee
Knee angle (°)	The sagittal plane angle between the thigh and lower leg segments (0° in the anatomical standing position, with positive values indicating flexion)
Ankle angle (°)	The sagittal plane angle between the lower leg and foot segments, calculated in a clockwise direction (110° in the anatomical standing position) (Cairns et al., 1986)

### Statistics

Results are reported as individual values or as means ± one standard deviation (SD). All statistical analyses were carried out using SPSS Statistics 27 (IBM SPSS, Inc., Chicago, IL). Because of the small sample size, Wilcoxon sign-rank tests were used to compare differences between laps for all variables; significance was set at *p* < 0.05, and effect sizes (*r*) were calculated using their magnitude as either small (0.10–0.29), medium (0.30–0.49), or large (≥ 0.50) (Field, 2009).

## Results

**Figure 2:**
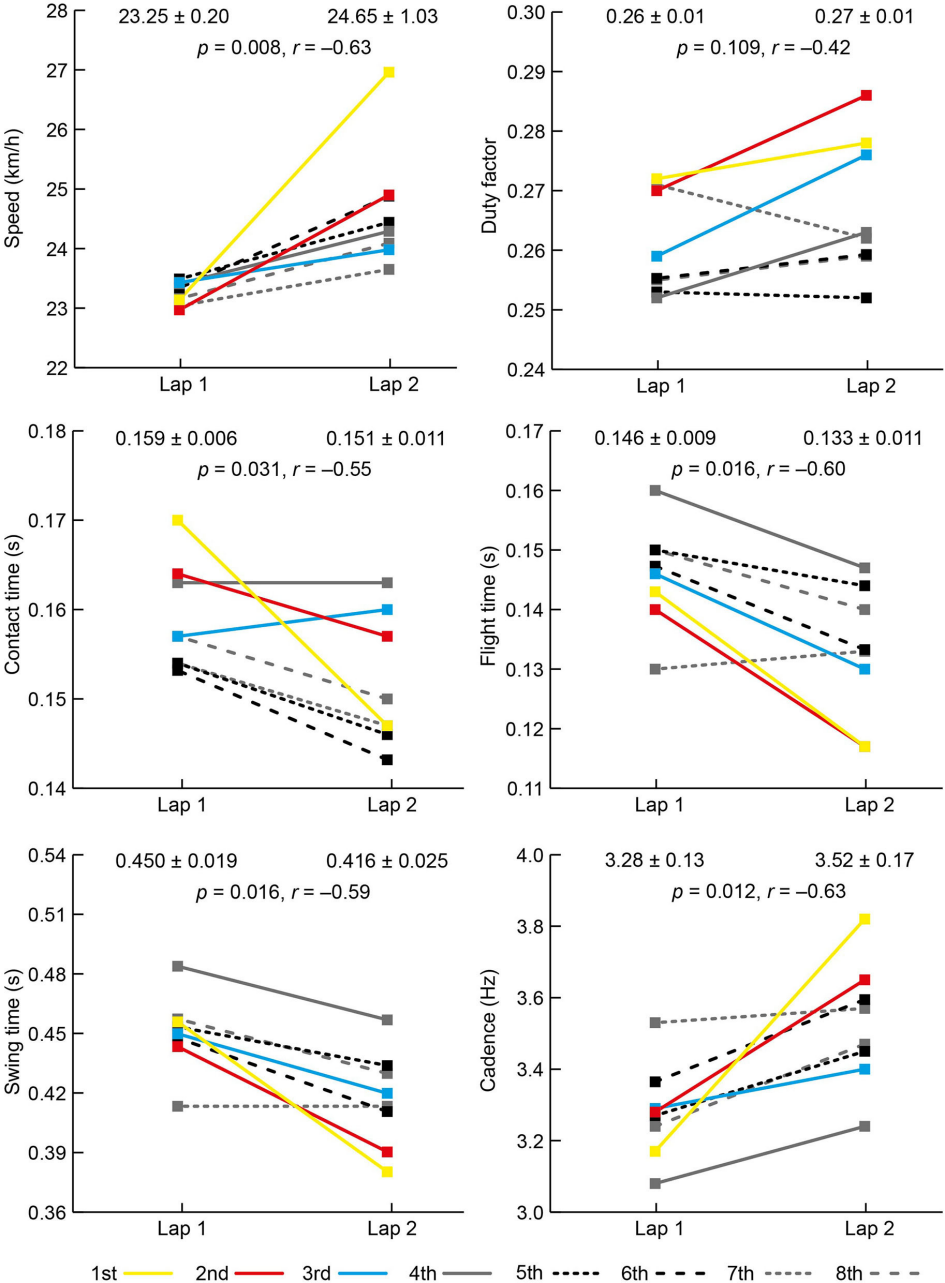
Mean (±SD) and individual athlete data for speed and temporal data on laps 1 and 2 of the women's 800 m final. The color used to represent each athlete is the same for each variable and the key for finishing positions is shown at the bottom of the figure.

**Figure 3:**
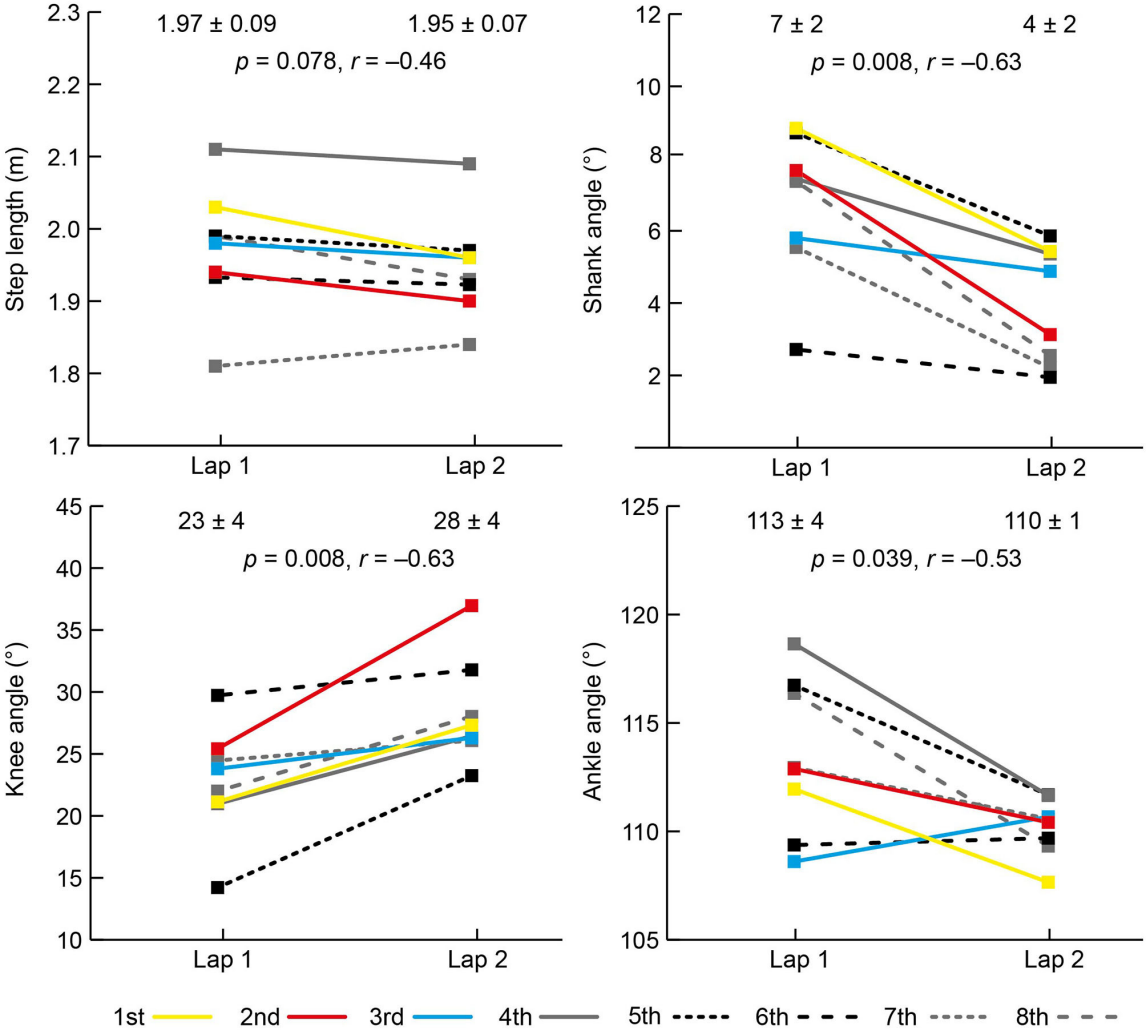
Mean (±SD) and individual athlete data for spatial and angular data on laps 1 and 2 of the women's 800 m final. The color used to represent each athlete is the same for each variable and the key for finishing positions is shown at the bottom of the figure.

The mean PB time (min:s) for the eight athletes before the race was 1:56.79 (±1.28) and their mean finishing time was 1:57.38 (±1.37), equivalent to 100.5% (±0.6) of PB time; two athletes set PB times during the final. The results for each spatiotemporal and joint kinematic variable on both laps are shown in Figures 2, 3. All athletes landed with a forefoot striking pattern on both laps.

There was an increase in speed between laps from 23.25 km/h (±0.20) to 24.65 km/h (±1.03) with all women faster on the second lap (Figure 2). There was an increase in cadence from 3.29 to 3.52 Hz (Figure 2), but there was no change in step length (Figure 3). Individually, all eight competitors had greater cadences on the second lap (Figure 2). As well as a decrease in contact time from lap 1 to lap 2, there was a decrease in flight time, itself caused by a decrease in swing time (Figure 2), but there was no change in duty factor. At initial contact, there was a 5° decrease in knee angle (more flexed) and a 3° decrease in ankle angle (more plantarflexed). The shank angle at initial contact also decreased from lap 1 to lap 2 (Figure 3).

## Discussion

The aim of this observational study was to analyze changes in spatiotemporal factors in world-class women's 800 m running between the end of the first and second laps. Although mean running speed on the first lap was relatively fast (equivalent to 2:03.8 pace), all women sped up on lap 2, showing that they were running faster even though they were likely to be tiring. The pacing profiles for this final (Hanley et al., 2018) showed that it was a tactical race, in line with other championship 800 m finals (Hanley et al., 2019), where athletes adopt a slower speed during the second half of the first lap, and attempt to maintain a fast sprint finish over the last 100 m. Such tactics mean that the results of this study are unique to this race, and will differ in other races where athletes adopt alternative tactics. The hypothesis that faster running speeds would result from increased cadence was supported; however, the hypothesis that step length would similarly increase was not supported. This shows that athletes rely on higher cadences for faster speeds and, on this most fundamental basis, athletes who wish to be successful 800 m runners must develop the neural adaptations required for higher cadence (Salo et al., 2011). All eight women increased cadence from lap 1 to lap 2, and the biggest increase (+ 21%) was for the eventual winner (to 3.82 Hz, equivalent to a rate of 229 steps/min) with the second biggest increase (+11%) for the silver medalist. Although it is not always the case that the fastest runners are those with the highest cadences (van Oeveren et al., 2021), increasing cadence when step length is already close to its maximum is clearly the principal factor in achieving world-class finishing speeds in competition. Such a high cadence is not feasible over the whole of the race, or over longer distance events, given its greater metabolic cost (Lieberman et al., 2015), and so is best left as the key biomechanical resource to tap into during the sprint for the finish line.

The temporal changes that occurred because of increased cadence were large reductions in contact time and flight time. The athletes achieved this critical reduction in flight time and a decrease in the time during swing (toe-off to initial contact) through higher cadences, which are largely achieved in fast running by flexing the hip faster (Bushnell and Hunter, 2007). It was noticeable in Figure 1 that the top two finishers had the shortest flight times on lap 2, resulting from their higher cadences, and ultimately a key factor in their success. Although lower duty factors are associated with faster running, they did not decrease between laps because the decrease in contact time was effectively offset by the decrease in swing time (and therefore total step time) and shows that duty factor is relatively constant because elite-standard athletes have robust mechanisms to exploit the advantages of better spring-mass behavior (Burns et al., 2021). Regardless of how the 800 m is a middle-distance event that relies on predominantly aerobic energy metabolism, the final stages are more sprint-like in mechanical terms and demonstrate the value of developing sprinting form in training (Bushnell and Hunter, 2007). Indeed, the winner of the women's 800 m ran a quicker time over the last 100 m (13.54 s) than the women competing over 400 m in the same championships (Hanley et al., 2018; Pollitt et al., 2018), and racing occasionally over 400 m could therefore be a coaching aid to coping with the demands of maintaining sprinting form while fatigued (Gamboa et al., 1996).

The 3° decrease in shank angle at initial contact and a knee that flexed 5° more at initial contact on lap 2 resulted from the higher cadences, and which might have been partly responsible for the decrease in swing time. Despite a consistent forefoot footstrike pattern (achieved through the ankle's plantarflexed position), the mean shank angle at initial contact was greater than zero (7° and 4° on laps 1 and 2, respectively), with the ankle therefore landing anterior to the knee. The shank angle at initial contact has been referred to previously as the “overstride angle” (Squadrone et al., 2015), which could give the impression that a touchdown position where the ankle is anterior to the knee is a negative feature of an individual runner's technique. By contrast, this leg position is clearly normal within this world-class sample of middle-distance runners. It might be that it is not the shank's orientation that affects running performance, but rather the landing foot's position relative to the CM that induces braking effects (Lieberman et al., 2015), and where too long a distance results in relatively long stance times (van Oeveren et al., 2021). Coaches should note that “overstriding” is a misleading term in describing foot landing position during running, regardless of which definition is used (Lieberman et al., 2015).

The most important strength of this novel research is that the athletes have been studied in a natural setting, providing high ecological validity. The results showed that the two laps are quite different (Gamboa et al., 1996), not only in physiological terms but also in biomechanical variables. As physiological conditioning is at its most discriminating influence with 200 m remaining (Hettinga et al., 2019), it is the ability of the athletes to adopt a metabolically costly but sprint-dependent increase in cadence. Indeed, during the endspurt, there were strong similarities in the values recorded for cadence and lower limb joint angles between the women's 400 and 800 m events (Pollitt et al., 2018). An inevitable limitation of collecting data in competition is that it is not possible to control the athletes' performances (as they can be in a laboratory), and so the tight bunching that occurred meant it was not possible to digitize obscured body parts (e.g., the arms) and analyze all potentially important variables. This means, for example, that measurements of CM position relative to foot position were not possible. It was also not possible to collect any physiological data that could have aided analysis, for example in relation to muscle activity. This study was limited by the small number of participants, and future research should attempt to capture more in-competition data that incorporate more middle-distance athletes (across abilities and sexes) to build a larger pool of data from which more advanced analyses are viable.

## Conclusions

In summary, this was the first study to analyze the women's 800 m at a major championships final across both laps. The athletes achieved faster running speeds during the sprint finish through increased cadence, which predominantly resulted in shorter flight times. Shorter swing times were part of the reason for these shorter flight times, as were a greater flexion of the knee and a greater plantarflexion of the ankle at initial contact. The well-established need for considerable anaerobic energy system training during the endspurt should be complemented by neural adaptation training that assists a high cadence, horizontal impulse production and spring-mass behavior (especially given the forefoot landing position adopted). The world's fastest runners have shank angles at initial contact greater than 0° at very fast running speeds, and the ubiquity of this landing position amongst this group shows that it is normal for the ankle to land anterior to the knee in middle-distance running. Although many 800 m athletes double up over 1,500 m, the 400 m event might also provide the opportunity to develop the sprint mechanics used in the endspurt despite fatigue. In this study, increasing cadence, rather than step length, was the driving factor for faster finishing speeds in the closing stages of the women's 800 m, and should be a key focus for athletes when training for 800 m racing.

## Data Availability Statement

The datasets presented in this article are not readily available because dataset has been part of a wider project commissioned by World Athletics. Requests to access the datasets should be directed to b.hanley@leedsbeckett.ac.uk.

## Ethics Statement

The studies involving human participants were reviewed and approved by Carnegie School of Sport Research Ethics Committee, Leeds Beckett University. The patients/participants provided their written informed consent to participate in this study.

## Author Contributions

AB and SM arranged data collection during the World Championships as Project Director and Project Leader, respectively. BH and AB performed data collection. BH processed the data and created the figure. All authors conceptualized and designed the study, wrote the manuscript, interpreted the results of the research, edited, critically revised, and approved the final version for submission.

## Funding

The data collection and initial data analysis were supported by funding provided by the IAAF/World Athletics as part of a wider development/education project; however, the nature of the data is purely descriptive and not associated with any governing body, commercial sector, or product. No funding was provided for the writing of this manuscript. The results of the present study do not constitute endorsement by the World Athletics.

## Conflict of Interest

The authors declare that the research was conducted in the absence of any commercial or financial relationships that could be construed as a potential conflict of interest.

## Publisher's Note

All claims expressed in this article are solely those of the authors and do not necessarily represent those of their affiliated organizations, or those of the publisher, the editors and the reviewers. Any product that may be evaluated in this article, or claim that may be made by its manufacturer, is not guaranteed or endorsed by the publisher.
